# Comparison of T2-preparation and magnetization-transfer preparation for black blood delayed enhancement

**DOI:** 10.1186/1532-429X-18-S1-Q10

**Published:** 2016-01-27

**Authors:** Elizabeth Jenista, David C Wendell, Han W Kim, Wolfgang G Rehwald, Stephen Darty, Enn-Ling Chen, Michele Parker, Raymond Kim

**Affiliations:** 1 Duke University, Durham, NC USA; 2 Siemens Healthcare, Chicago, IL USA

## Background

A fundamental component of the CMR exam is contrast enhanced imaging, which is crucial for delineating diseased from normal tissue. Unfortunately, diseased tissue immediately adjacent to blood often is hidden since there is poor contrast between hyperenhanced tissue and bright blood. A new method recently described, **F**low-**I**ndependent **D**ark-blood **D**e**L**ayed **E**nhancement technique (FIDDLE), allows visualization of tissue enhancement while suppressing blood signal. One critical part of FIDDLE is the prep pulse prior to inversion, which accentuates differences in magnetization between tissue and blood. In this study, we compared a T2-prep and a magnetization transfer (MT) prep for use with FIDDLE.

## Methods

The components of FIDDLE are, (1) a prep pulse that differentially saturates tissue compared with blood (e.g. MT or T2); (2) phase-sensitive inversion recovery (PSIR); and (3) inversion time (TI) selection under condition: blood M_Z_ < tissue M_Z_. T2-prep or MT-prep FIDDLE were compared in a canine model of MI (n = 9) and in MI patients (n = 3). The two sets of FIDDLE images were acquired in an interleaved fashion 10-20 mins after gadolinium administration (0.2 mmol/kg) using identical parameters. Images were visually graded for overall quality, and for visible blood pool artifacts. Additionally, images were analyzed by placing ROIs in the ventricular and atrial blood pool, and over the entire heart (blood pool and myocardium in all chambers). The magnitude of blood pool artifacts were calculated by assessing the percent variation in blood pool signal and normalizing this to the standard deviation of the signal from the entire heart.

## Results

**Figure 1:**
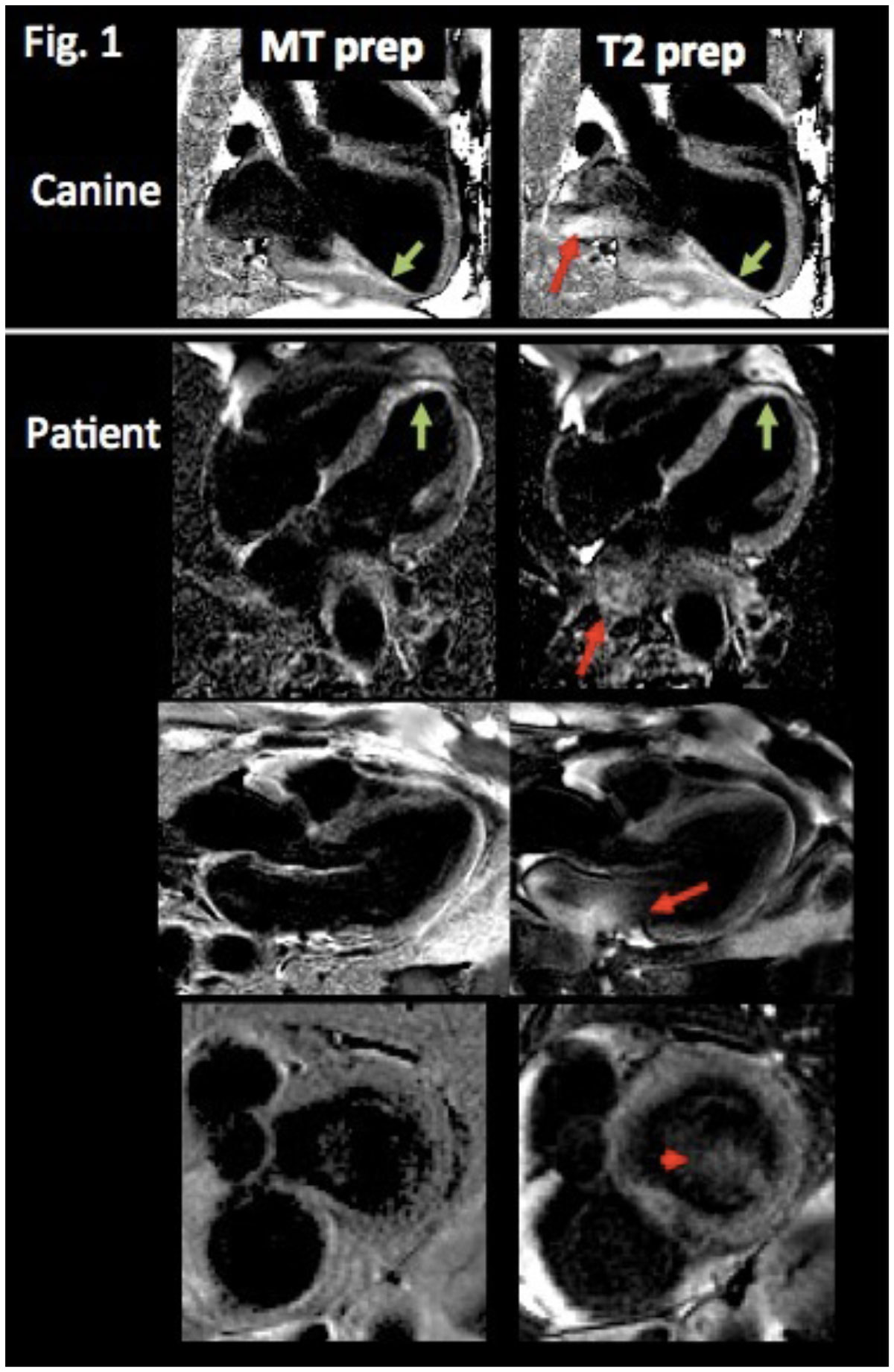
Figure 1

On visual assessment, FIDDLE was effective in suppressing ventricular blood signal, which allowed differentiation of subendo MI from blood (Figure 1**green arrows**). In contradistinction, often there was non-uniform blood suppression in the left atrium (LA) with T2 FIDDLE, which was not seen with MT FIDDLE (**red arrows**). 57% (8/14) and 91% (10/11) of T2 FIDDLE showed LA blood artifacts in canines and patients, respectively, whereas none of the MT FIDDLE showed artifacts (both p < 0.05). The LA blood artifacts appeared to arise predominately in areas of fast blood flow at the edge of the field-of-view, e.g. regions of in-flow from pulmonary veins. Occasionally the artifact extended from the LA into the basal portion of the LV (**red arrowhead**). Quantitative assessment verified that both methods provided similar homogeneity for LV blood pool whereas the LA blood was significantly more inhomogeneous with T2 than with MT FIDDLE (Figure 1).

## Conclusions

This study demonstrates that both T2-prep and MT-prep can be employed to produce black-blood delayed enhancement images. MT FIDDLE provides uniform blood suppression throughout ventricular and atrial blood pools whereas there was often inhomogeneous blood signal in the LA for T2 FIDDLE.

**Figure 2:**
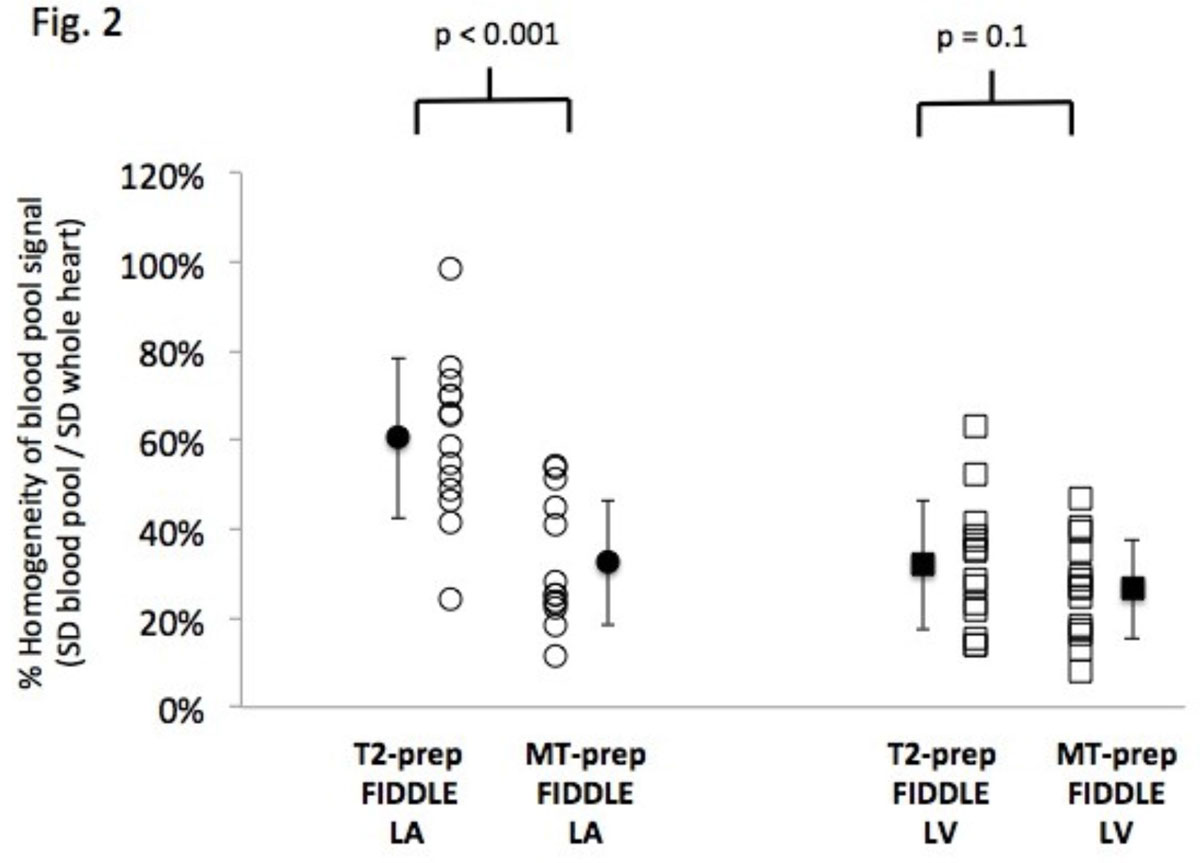
Figure 2

